# Economic games and social neuroscience methods can help elucidate the psychology of parochial altruism

**DOI:** 10.3389/fpsyg.2015.00861

**Published:** 2015-07-07

**Authors:** Jim A. C. Everett, Nadira S. Faber, Molly J. Crockett, Carsten K. W. De Dreu

**Affiliations:** ^1^Department of Experimental Psychology, University of OxfordOxford, UK; ^2^Oxford Martin School, University of OxfordOxford, UK; ^3^Department of Psychology, University of AmsterdamAmsterdam, Netherlands; ^4^Center for Research in Experimental Economics and Political Decision Making, University of AmsterdamAmsterdam, Netherlands

**Keywords:** parochial altruism, ingroup favouritism, preferences, beliefs, economic games, oxytocin, testosterone

The success of *Homo sapiens* can in large part be attributed to their highly social nature, and particularly their ability to live and work together in extended social groups. Throughout history, humans have undergone sacrifices to both advance and defend the interests of fellow group members against non-group members. Intrigued by this, researchers from multiple disciplines have attempted to explain the psychological origins and processes of *parochial altruism*: the well-documented tendency for increased cooperation and prosocial behavior within the boundaries of a group (akin to ingroup love, and ingroup favoritism), and second, the propensity to reject, derogate, and even harm outgroup members (akin to “outgroup hate,” e.g., Tajfel and Turner, 1979; Brewer, 1999; Hewstone et al., 2002; Choi and Bowles, 2007; De Dreu et al., 2014; Rusch, 2014). Befitting its centrality to a wide range of human social endeavors, parochial altruism is manifested in a large variety of contexts that may differ psychologically. Sometimes, group members help others to achieve a positive outcome (e.g., gain money); and sometimes group members help others avoid a negative outcome (e.g., avoid being robbed). Sometimes, group members conflict over a new resource (e.g., status; money; land) that is currently “unclaimed”; and sometimes they conflict over a resource that is already held by one group.

In this paper, we take stock of exciting new directions and methods in the psychological study of parochial altruism. We argue that to enrich our understanding of the psychological processes underlying parochial altruism, researchers could (continue to) incorporate methods and insights developed and popularized in adjacent disciplines, such as behavioral economics and social neuroscience. First, we highlight how the discipline of behavioral economics and its associated methodology of *economic games* can enrich our psychological understanding of parochial altruism through exploring the manifestation of, and psychological mechanisms driving, parochial altruism in both gains and losses contexts. Second, we consider the social neuroscientific approach, highlighting how research into neuromodulators has advanced our understanding of parochial altruism by outlining differential influences of the neuromodulators testosterone and oxytocin on ingroup cooperation and outgroup discrimination. Given that parochial altruism is at root an interdisciplinary phenomenon, it would be a pity if each discipline that studies it does so from and within its own silo. With greater incorporation of these new directions in parochial altruism, scientists can enrich their understanding as to when, why, and how people help members of their own group more than other groups, and even harm members of other groups.

## Economic games: Structuring conflict and cooperation

In recent years, the study of parochial altruism—in evolutionary biology, behavioral economics, and social psychology—has increasingly drawn on the methodology of economic games, inspired by both psychological research (e.g., Tajfel, 1970; Pruitt and Kimmel, 1977; Komorita and Parks, 1995) and behavioral game theory (e.g., Camerer, 2003). In a conscious tradeoff, researchers using economic games sacrifice the real-world validity of field-based studies in social psychology and opt instead for tightly controlled experiments that test how people make incentive-compatible choices concerning resource distribution. The core feature of economic games is their simplicity, where one player usually has a strictly dominant strategy if they are self-interested, and where this selfish strategy is salient and easy to understand in all cases. If and when a player does not choose this selfish strategy we can infer that they did not do so because they had some other motive (e.g., Deutsch, 1949; Messick and McClintock, 1968; Fehr et al., 2006). This possibility to being able to draw strong inferences about the extent to which individual sacrifice immediate self-interest is a clear advantage offered by economic games, relative to some other paradigms used to study intergroup discrimination and parochial altruism.

Within this approach, the conceptual apparatus of preferences and beliefs is particularly useful for explaining intergroup prosocial behavior (Everett et al., 2015). Preferences refer to a person's tendency toward certain behaviors and outcomes in a given context based on the expected utility to be derived from them, while beliefs refer to the expectations that people have about uncertain outcomes in a game (Camerer, 2003). In any given context, preferences and beliefs can either promote or hinder prosocial behavior. Economic games differ in the extent to which they measure both general and specific preferences and/or beliefs. In some games, behavior can be explained primarily by social preferences—for example, preferences involving fairness (e.g., the *Dictator Game*: Kahneman et al., 1986), or preferences to either help ingroup members or to also (or even exclusively) harm outgroup members (e.g., *Intergroup Prisoner's Dilemma—Maximizing Difference*: Halevy et al., 2012; Buttelmann and Böhm, 2014). In other games, behavior seems driven primarily (but often not exclusively) by beliefs—for example beliefs regarding trustworthiness (e.g., the *Trust Game*: Berg et al., 1995), reciprocity (e.g., Public Good Games: e.g., De Cremer and Van Vugt, 1999; Fischbacher et al., 2001), or expectations of norm enforcement (e.g,. the *Ultimatum Game*: Güth et al., 1982). But to what extent is parochial altruism driven by preferences and beliefs? Economic games allow researchers to address this question not only by teasing apart dominant processes in explaining parochial altruism, but also by elucidating moderating conditions (e.g., whether a decision is public or private; or whether the behavior is costly or cheap). A central advantage of economic games is that their simple structure allows small modifications that change some psychologically relevant feature while preserving the incentive and payment structure. For example, researchers can manipulate whether games are played publicly or anonymously (e.g., Yamagishi and Mifune, 2008), whether games are repeated or played just once (e.g., Gächter et al., 2010), or whether the games are played with artificial or real groups (e.g., Jackson, 2008).

One example of how using economic games has the potential to elucidate basic psychological mechanisms operating in parochial altruism comes from the consideration of loss aversion. For example, most experimental research on parochial altruism has looked at situations in which a participant helps an ingroup (vs. outgroup) member gain something positive. Yet one of the most established findings in cognitive psychology—loss aversion—is that people are more sensitive to losses than gains such that people strongly prefer avoiding losses than achieving gains (Kahneman and Tversky, 1979). Research suggests that because inflicting a loss is seen as more harmful and fairness-violating than withholding a gain, individuals are more likely to help another avoid experiencing a harmful outcome than they are to help provide a positive outcome (De Dreu and Kret, 2015). Moreover, in an intergroup context, outgroup hate is typically manifested as the absence of helping, rather than inflicting harm (Mummendey and Otten, 1998; Weisel and Böhm, 2015). But how might specifically ingroup-favoring prosocial behavior be differentially manifested in losses vs. gains context? This remains an open question, for almost no research has examined the effects of gains and losses in specifically intergroup contexts, nor the extent to which ingroup favoring prosocial behavior is driven by the same preference or belief-based psychological processes in gains and losses contexts. Given the centrality of parochial altruism to any psychological or evolutionary discussion of prosocial behavior and morality, this constitutes an exciting opportunity for future research. For example, it might be predicted that because fairness concerns are more salient in interactions with ingroup members than with outgroup members, and because fairness concerns are more prominent in loss contexts than in gain contexts, that people might show greater ingroup favoritism in loss contexts.

## Neuromodulation of parochial altruism

Social neuroscience has already made substantial advances in our understanding of parochial altruism, particularly in elucidating whether and how different brain regions, such as the amygdala and the prefrontal cortex are associated with group-related behavior (for recent reviews see Molenberghs, 2013; Amodio, 2014; Baumgartner et al., 2014; Cikara and Van Bavel, 2014). Comparatively little work has focused on how specific neuromodulators underlie parochial altruism, even though this could be a promising new direction to understand parochial altruism. If parochial altruism has fitness functionality that explains its evolution (Rusch, 2014) such that humans are biologically prepared for parochial altruism, then humans may have neuromodulatory systems that regulate this behavior. Consequently, understanding these neuromodulatory systems can contribute to the understanding of intergroup prosocial behavior. Here, we discuss the role of two neuromodulators in parochial altruism, oxytocin and testosterone.

### Oxytocin

Recent work has implicated the neuropeptide *oxytocin* in parochial altruism (De Dreu et al., 2014; De Dreu and Kret, 2015). Oxytocin has a range of effects on the brain, body, and behavior, but here we focus on two of its psychological effects: reducing anxiety and fear of betrayal, and up-regulating positive regard for others (De Dreu and Kret, 2015).

To the extent that oxytocin is a neurohormonal system involved in parochial altruism, pharmacological studies involving administration of oxytocin to participants playing economic games should give insight into the biological and psychological processes underlying parochial altruism. Directly testing whether oxytocin influences ingroup love or outgroup hate, De Dreu et al. (2010) looked at behavior in economic games (the Intergroup Prisoner's Dilemma—Maximizing Difference and between-group Prisoners Dilemmas) under conditions of intranasal oxytocin administration vs. placebo. They found that across three experiments, individuals administered oxytocin displayed more ingroup trust and ingroup love, but did not display more outgroup hate and outgroup distrust, relative to the placebo condition. In a follow-up, De Dreu et al. (2012) took advantage of advantages of the possibility offered by economic games to make simple changes to the structure of the intergroup conflict game so as to elucidate whether oxytocin-modulated outgroup competition was motivated by a desire to protect oneself and/or fellow group-members against high threat outgroups. Results again showed that oxytocin-modulated parochial altruism was driven by a “tend-and-defend” functionality—under oxytocin individuals display more ingroup love and stronger tendency to aggressively protect oneself and fellow group members against threatening outsiders. In addition, and of key relevance here, is that this work, and the specific methodological approach of combining advantages of economic games and social neuroscience methods, can provide new and exciting insights into both psychological and biological mechanisms underlying parochial altruism.

### Testosterone

A second neuromodulator that has been implicated in parochial altruism is testosterone. Testosterone is a steroid hormone that is secreted in mammals in the male testes and, to a lesser extent, the female ovaries. Testosterone has been shown to be associated with reduced trust (Bos et al., 2012), vicarious experiences of group success (Bernhardt et al., 1998), status seeking (Eisenegger et al., 2011), dominance (Mehta and Josephs, 2010), and aggression (see Montoya et al., 2012 for a review).

Comparatively little research has explored the role that testosterone plays in parochial altruism specifically, yet it seems likely that testosterone—like oxytocin—plays an important role. Consider, for example, a recent study by Diekhof et al. (2014), who had fifty male soccer fans with a strong group identity respond to Ultimatum Game (UG) offers that were either fair or unfair, and proposed either from an ingroup member or by fans of one of three other teams (two soccer teams and one cricket team). In the UG, one player makes a proposal to the other player (the responder) for how to divide a pool of money between them. If the responder accepts the proposed split, both players receive the allocated money. However, if the responder rejects the proposed split, both players receive nothing. Behavior in the UG is typically seen as reflecting norm enforcement of “fair” allocations. Results showed that unfair offers were rejected more frequently than fair offers, and that overall rejection rates increased with social distance to the outgroups (ingroup < neutral outgroup < unknown outgroup < antagonist outgroup). Furthermore, endogenous testosterone was associated with lower rejection of ingroup offers and with increased rejection of outgroup offers—especially in the context of explicit intergroup competition. High endogenous testosterone, then, underlies parochial altruism through increased prosocial tendencies during interactions with the ingroup, as well as through an escalation of costly outgroup hostility in intergroup competition. Apart from advancing our knowledge of how testosterone underlies parochial altruism, such work highlights the way in which ingroup love and outgroup hate are distinct but complementary psychological processes explaining parochial altruism. Moreover, such work highlights the importance of the context in which the intergroup prosocial behavior takes place: oftentimes it is not merely the presence of group members that leads to parochial altruism, but rather the existence of intergroup competition. As with oxytocin, it would be fruitful for future research to explore further this relationship by examining the way in which administration of testosterone influences ingroup love and outgroup hate in economic games.

## Conclusion

Parochial altruism is a complex and interdisciplinary phenomenon, and so it stands to reason that to understand parochial altruism we should turn to a range of different techniques from a range of disciplines. Economic games are especially useful due to their isolation of distinct preference and belief-based psychological processes (e.g., Everett et al., 2015) and the way in which they can be easily accommodated with other methods, such as pharmacological manipulations, that highlight how humans are biologically prepared for parochial altruism and have neuromodulatory systems that regulate this behavior (e.g., De Dreu and Kret, 2015). Through greater incorporation of different methodologies and perspectives—such as the use of economic games—, researchers can come to a more complete understanding of the psychological processes that explain how, when, and why people help members of their own group more than others.

## Author note

JE drafted the paper and NF, MC, and CD provided comments and suggested revisions.

### Conflict of interest statement

The authors declare that the research was conducted in the absence of any commercial or financial relationships that could be construed as a potential conflict of interest.
